# Demonstration of Genome-Wide Association Studies for Identifying Markers for Wood Property and Male Strobili Traits in *Cryptomeria japonica*

**DOI:** 10.1371/journal.pone.0079866

**Published:** 2013-11-19

**Authors:** Kentaro Uchiyama, Hiroyoshi Iwata, Yoshinari Moriguchi, Tokuko Ujino-Ihara, Saneyoshi Ueno, Yuriko Taguchi, Miyoko Tsubomura, Kentaro Mishima, Taiichi Iki, Atsushi Watanabe, Norihiro Futamura, Kenji Shinohara, Yoshihiko Tsumura

**Affiliations:** 1 Department of Forest Genetics, Forestry and Forest Products Research Institute, Tsukuba, Ibaraki, Japan; 2 Laboratory of Biometry and Bioinformatics, Department of Agricultural and Environmental Biology, Graduate School of Agricultural and Life Sciences, The University of Tokyo, Bunkyo, Tokyo, Japan; 3 Graduate School of Science and Technology, Niigata University, Niigata, Japan; 4 Forest Tree Breeding Center, Forestry and Forest Products Research Institute, Hitachi, Ibaraki, Japan; 5 Faculty of Agriculture, Kyushu University, Fukuoka, Fukuoka, Japan; 6 Department of Molecular and Cell Biology, Forestry and Forest Products Research Institute, Tsukuba, Ibaraki, Japan; 7 Forestry and Forest Products Research Institute, Tsukuba, Ibaraki, Japan; Nanjing Forestry University, China

## Abstract

Genome-wide association studies (GWAS) are an alternative to bi-parental QTL mapping in long-lived perennials. In the present study, we examined the potential of GWAS in conifers using 367 unrelated plus trees of *Cryptomeria japonica* D. Don, which is the most widely planted and commercially important tree species in Japan, and tried to detect significant associations between wood property traits and quantity of male strobili on the one hand, and 1,032 single nucleotide polymorphisms (SNPs) assigned to 1,032 genes on the other. Association analysis was performed with the mixed linear model taking into account kinship relationships and subpopulation structure. In total, 6 SNPs were found to have significant associations with the variations in phenotype. These SNPs were not associated with the positions of known genes and QTLs that have been reported to date, thus they may identify novel QTLs. These 6 SNPs were all found in sequences showing similarities with known genes, although further analysis is required to dissect the ways in which they affect wood property traits and abundance of male strobili. These presumptive QTL loci provide opportunities for improvement of *C. japonica*, based on a marker approach. The results suggest that GWAS has potential for use in future breeding programs in *C. japonica*.

## Introduction

The genetic improvement of forest trees is greatly limited by their long lifespan, large plant size, extended juvenile phase of seedlings, and the fact that most agronomically-important traits such as wood property cannot be assessed until a seedling has matured physiologically [1]. The long generation times required for completing a breeding cycle, the costs involved in establishing and maintaining large progeny trials, and the low heritability of most target traits are further factors which have impeded progress in the genetic improvement of these species. Marker-assisted selection (MAS) is one technology which may help to surmount the barriers to forest tree breeding because it enables selection without field testing and can therefore accelerate the selection process, and make it possible to reduce the numbers of progeny required and the costs of growing individuals to maturity in the field. In spite of the potential advantages of MAS for forest trees, it was initially dismissed as a realistic approach for most operational tree breeding programs, and its application has been limited to the selection of a few simply inherited traits [2], because marker development for MAS via bi-parental quantitative trait loci (QTL) mapping is hindered by the same complications as described above. Moreover, the majority of QTL mapping studies reported in the literature are based on bi-parental mapping populations and designed to identify QTLs that co-segregate with phenotypic traits within a bi-parental family. The inferences possible from bi-parental-based QTL mapping are limited to the particular parents used to generate the mapping population, i.e. to the genetic makeup of these parental lines combinations thereof. Extrapolation beyond the original mapping population will likely be invalid due to the lack of knowledge of identity by descent at a specific genomic region [3]. To be able to identify and exploit QTLs from a broad range of germplasm, including elite lines and wild ancestors, it is necessary for mapping to be expanded beyond a bi-parental base.

Recently, genome-wide association studies (GWAS) have been increasingly used for detecting important genes related to traits of interest, especially in model and other important organisms [4]–[6]. GWAS approaches have several advantages over bi-parental QTL mapping: (i) GWAS does not require the development of a specific segregating population to detect QTL, (ii) GWAS can explore QTL controlling variations in a much larger and more representative gene-pool without any prior information about candidate genes, and (iii) GWAS is considered to provide much higher resolution than bi-parental QTL mapping, resulting in narrow confidence intervals for the loci detected. In some cases, historic phenotyping data for a number of important traits is already available from conventional breeding programs. Such data can potentially minimize the time and cost associated with phenotyping. Association genetics in forest trees has been proposed as an efficient way of overcoming the intrinsic limitations of QTL-based MAS [7].

Coniferous species have a large genome size (more than 10 Gb; [8]), and thus most of them have a large genetic-to-physical distance ratio (>3000 kb/cM). This makes it difficult to identify the genes responsible for phenotypic variations using GWAS, because linkage disequilibrium (LD) in coding regions rapidly decays within a short distance (a few thousand bp; [7]). For this reason, association studies based on pre-selected candidate genes have been applied more widely than GWAS in conifers [9]–[12]. The challenge of genotyping a large number of individuals using a large number of genome-wide markers is another factor which has restricted the application of GWAS in conifers up to now (but see [13]). The development of high-throughput systems such as next generation sequencing and SNP arrays, however, should make it possible to overcome this difficulty, since they provide highly multiplexed platforms, which allow rapid and cost-effective genotyping over a massive number of SNPs.

Even though high-throughput genotyping systems open up a new approach to association genetics, several factors may influence the power with which true associations can be detected [7], [14], [15]. These factors can often be accounted for at the experimental design stage, by taking into account the extent of genome-wide LD, the number of genes affecting the trait, allele frequency, and sample size. The use of natural populations or pedigrees for association studies can introduce confounding genetic structure [16], [17], which can create false LD between markers and QTL [7]. Although conifers generally have low levels of population structure, these species have grown under fluctuating environmental conditions for long periods and they display clear phenotypic adaptations to environmental gradients at multiple spatial scales [18]. Years of provenance, common garden, and genecological studies have unveiled the highly polygenic basis of these adaptive traits [19], suggesting that it is essential to incorporate population structure effects in association analysis of coniferous species.

In the present study, we investigated the potential of GWAS in *Cryptomeria japonica*, which is the most important forestry tree species in Japan. The species has been planted widely throughout Japan and it currently covers an area of 4.5 million ha, accounting for 44% of all Japanese artificial forests. Fifteen million seedlings are supplied as material for afforestation every year, so that this species is as important for Japanese forestry now as it has been since ancient times. Modern natural forests of *C. japonica* are distributed across a range of different environments in the Japanese Archipelago, from Aomori Prefecture (40° 42′ N) to Yakushima Island (30° 15′ N) [20]. Geographical variation between natural forests of *C. japonica* has been investigated, focusing on morphological traits (needle length, needle curvature, and other features; [21]), diterpene constituents [22], and DNAs [23], [24]. The results of these studies suggest that there are two main types, i.e., Ura-sugi (*C. japonica* var. *radicans*, found near the Sea of Japan) and Omote-sugi (*C. japonica*, located near the Pacific Ocean), which are thought to have adapted to the contrasting environmental conditions found on the two sides of the Japanese Archipelago. Since 1960, more than 3,700 elite trees have been selected as first generation plus trees, mainly from the artificial forests (some trees selected from natural populations have also been included), to be used as the basis for forest planting and breeding. Although elite trees can be good candidates for GWAS, the extent of LD and genetic stratification, which are factors affecting the power of GWAS, in the elite tree population have not been investigated at a genome-wide level.

In the present study, we focused on wood property and quantity of male strobili in GWAS. In *C. japonica* breeding programs using elite trees as starting material, wood property is one of the main breeding targets. To our knowledge, however, no candidate gene underlying this trait has yet been detected. Because it takes a long time to evaluate wood property in conventional breeding programs, markers that facilitate selection of trees with high wood quality will have a major impact on *C. japonica* breeding. In addition to wood quality, pollen-related characteristics such as male sterility and low pollen fecundity are other important breeding targets in *C. japonica*, because allergic reactions to pollen of this species have recently become a severe public health problem in Japan. A nationwide epidemiological survey found that at least 26.5% of the Japanese population suffers from pollinosis due to *C. japonica* pollen [25]. To address this problem, several approaches have been attempted by local forest research institutes and the Forestry Agency in Japan. These groups have developed and made available male-sterile individuals and individuals with low pollen fecundity. Thus, the quantity of male strobili is also a strong candidate as a trait for MAS.

Here, we use genome-wide marker data to address the following questions: (1) What are the patterns of population structure and LD in first-generation plus trees of *C. japonica*? (2) Can the GWAS approach be effective in the plus tree population of *C. japonica*? (3) What is the extent of confounding between QTLs and population structure due to geography? To answer these questions, we evaluate the extent of LD and population structure in plus trees by comparison with samples taken from the entire distribution range of *C. japonica*, adopting a mixed linear model for GWAS which takes into account kinship relationships and subpopulation structure among the plus trees. Finally, the potential of GWAS and the prospects for the application of GWAS in *C.japonica* breeding programs are discussed.

## Materials and Methods

### Plant Materials

Since 1960, more than 3,700 plus trees have been selected, mainly from artificial forests (some trees have also been selected from natural populations), to use as the starting material for forest planting and breeding. The selection process has targeted high tree growth rate, narrow tree crown and other superior traits [26] but not for wood quality and male flower fecundity as target traits in our analysis so there might be no diversity losses due to selection for these traits. Based on genotype data obtained using microsatellite markers (Miyamoto *et* *al*, in preparation), we chose for GWAS 367 unrelated genotypes of *C. japonica* plus trees which were selected from artificial stands of national forests in the Kanto Breeding Region which is located in the eastern part of Japan (no specific permissions were required because of national forests). All trees were propagated by grafting in the Okubo Breeding Stock Garden 36°33′ N, 140°36′ E) at Hitachi, Ibaraki Prefecture with three replicates. The genetic structure within this region is believed to be relatively weak or unclear compared to that across the entire distribution range for this species [23]. Therefore, for population structure analysis, we used two additional sets of samples of *C. japonica* to represent its complete range: one set was taken from 14 natural populations (7 populations each from Japan Sea side and Pacific Ocean side, n = 101 and 80, respectively) (n = 181) which locate in national forests [27] and the other consisted of core collection samples (n = 456) which were selected from over 3,700 plus trees on the basis of geographical, environmental, and genetic factors for constructing core collection of *C. japonica* (Miyamoto *et* *al*, in preparation). Of the 456 core collection samples, 113 were included in plus tree samples of Kanto Breeding Region.

### Phenotypic Data

#### Measuring wood property

Data on wood property were obtained from Mishima *et* *al* [28], and the method used and its implementation are described fully in that paper. The wood property of each clone with three replicates were evaluated in a 14-year-old of *C. japonica* stand at Okubo Breeding Stock Garden at Hitachi, Ibaraki, Japan (36°33′ N, 140° 36′ E), using stress-wave propagation methods [29], [30]. The applicability of vibration modes for assessing the quality of wood materials has been investigated extensively in recent years [30]–[33]. The stress wave velocity (SWV) through the stem of each tree was measured using a TreeSonic (Fakopp Enterprise, Hungary). Transmitter- and receiver-probes were inserted 1 m apart in the sapwood of the tree stem, and a stress wave was induced by means of a hammer impact. The transit time of the wave between the two probes was recorded electronically and was used to calculate the stress wave velocity. The magnitude of the wave velocity and the calculated dynamic modulus of elasticity (MOE) were used as indices for assessing wood property in standing trees. Three measurements were taken at breast height on two side surfaces of each tree. The data of stress wave velocity (as wood property in the present study) of our samples were clearly normally-distributed. Estimates of the broad-sense heritability of wood quality in *C. japonica* were 0.82 [34].

#### Measuring numbers of male strobili

Details of the measurement of male strobili numbers are given in Tsubomura *et* *al* [35]. Here, we give a brief summary of the measurement process. The abundance of male strobili for each clone with three replicates were evaluated using a grading score on an ordinal scale of 1 to 5, to represent the number of male strobili (1: no or few male strobili, 2: sparse male strobili on few branches, 3: sparse male strobili on about 50% of branches, 4: many male strobili over 50% of branches, 5: abundant male strobili on almost all branches) at the crossing garden of the Forest Tree Breeding Center, Forestry and Forest Products Research Institute, located in Hitachi, Ibaraki, Japan (36° 69′ N, 140° 69′ E, elevation: 52 m). The scoring was conducted by 9 different persons in December 2009. All observed branches had previously been sprayed (at the beginning of July 2009) with 100 ppm gibberellin solution to induce flowering. The mean score of the replicates for each clone after excluding the highest and lowest values of each ramet was taken used as the trait value for that clone. The data of male flower were also clearly normally-distributed [35]. The narrow-sense heritability of male flower production were ranging from 0.78 to 1.05 [36].

### Genotype Data

Genotyping was conducted with the Illumina GoldenGate SNP genotyping platform [37], [38]. This high-throughput platform works well for the large and complex genomes of conifers [39], [40]. We selected one SNP from each of 1,536 sequences, making the assumption that SNPs within different putative unigenes would be independently related to different genes. Criteria for selection of the SNPs were based on the Illumina design score (above 0.6). When multiple SNPs were available within the same sequence, only one, highly polymorphic, SNP was selected as a tag for the sequence [41]. The identification of SNPs was carried out in previous study through the resequencing of unique EST contigs using a discovery panel of four *C. japonica* individuals with different genetic background [41]. DNA was extracted from young leaves using a modified CTAB protocol [42], and standardized to Illumina-specified concentrations for SNP genotyping (50–250 ng/µl). Multiplexed genotyping was carried out according to the manufacturer’s protocol [37], [38]. All polymorphic primer sequences and NCBI accession numbers are available for download at ForestGen database http://www.ffpri.affrc.go.jp/labs/cjgenome/HTML/SugiSNP_primers.html).

Signal intensities were quantified and matched to specific alleles, using the Genome Studio v2010.2 software package (Illumina Inc., San Diego). The quality of GoldenGate genotype scores for individual SNPs was assessed from their GenTrain cluster and GenCall genotype scores in GenomeStudio. These scores reflect the degree of separation between homozygote and heterozygote clusters for each SNP. A minimum GenCall50 (GC_50_) score of 0.25 was chosen as the threshold for inclusion of SNP loci in the final data set, and genotypic clusters were edited manually when necessary. Of the 1,536 SNPs of our assay, 1,032 were polymorphic within the plus tree population from the Kanto Breeding Region.

### Population Structure

Population structure is the leading cause of false positives in genetic association studies. To examine potential population stratification in the 367 plus trees, we performed a STRUCTURE analysis [43] using 292 selected SNPs. These SNPs, which were roughly evenly spaced across the *C. japonica* genetic map (>1 cM between SNPs) [44], were subjected to stratification analyses [45]. We excluded SNPs that departed significantly from Hardy-Weinberg equilibrium, based on Fisher exact tests implemented in the GenePop software package [46]. For comparison, 14 natural populations (n = 181) and 456 core collection individuals of *C. japonica* (Miyamoto *et* *al*, in preparation) that covered the natural distribution range of the species were also analyzed. The admixture model and the linkage model were used in order to compare the results obtained using different models and to test the robustness of the results. Ten independent runs of K = 1–10 with 100 000 Markov chain Monte Carlo (MCMC) iterations and a burn-in period of 40 000 iterations were performed, assuming correlated allele frequencies [47]. Firstly, we analyzed the 14 natural populations and identified typical individuals for each cluster to use as reference points. Individuals with membership coefficients of q_j_≥0.8 were assigned to a specific group, whereas individuals with q_j_<0.8 were identified as admixed (if not otherwise specified). To assist in clustering, these samples from the natural subpopulations were also incorporated into the analyses of plus trees and taken as reference points using the USEPOPINFO option implemented in STRUCTURE [43]. The optimal value of K was defined as the one at which the log likelihood of the data, ln P(X|K) [43] or ΔK, the rate of change of ln P(X|K) between successive K values [48], was maximal. Results were summarized in matrices of fractional subpopulation membership (Q matrices).

### Linkage Disequilibrium

The level of LD and its significance for each pair of SNP loci on the same chromosome were evaluated using the GENETICS package (http://cran.r-project.org/web/packages/genetics/index.html) in R [49] with a significance threshold of P<0.01 and a False Discovery Rate (FDR) [50] corrected threshold (0.1) was applied. LD was estimated both for the entire population and for specific subgroups (membership coefficients of q_j_≥0.8). For comparison, we also estimated LD for the core collection samples. LD was estimated by computing the squared correlation coefficient (*r* ^2^) between pairs of markers and the significance was computed with 1000 permutations. The *r* ^2^ value was estimated both for unlinked loci and for loci on the same chromosome. The latter value was plotted against the genetic distance in centimorgans (cM) [44].

### Association Analyses

Association analysis was performed with the EMMA (efficient mixed model analysis) package [51] in R, based on a mixed model (MLM) approach accounting for population structure and kinship relatedness [52], [53]. Marker-based kinship was estimated as suggested by Habier *et* *al* [54]. To adjust significance levels for multiple testing [55], we constrained the FDR [50] to be less than 0.05, using the p.adjust function in R. To determine the influence of population stratification and kinship relationships among samples, we compared three models and examined the distribution of *P*-values obtained in the association tests: a mixed model with no structure or kinship effects (naïve model), with covariates to account for population structure (Q model) and a mixed model that incorporated both population structure and marker based kinship estimates (Q+K model). To make the comparisons, we plotted expected *P*-values against observed *P*-values following a uniform distribution between 0 and 1 [56]. The percentage of variance explained by each SNP was obtained using the kinshipBLUP function in rrBLUP [57]. Putative functions for the genes underlying the QTL loci were identified by performing BLASTx [58] similarity searches against the National Center for Biotechnology Information (NCBI) nonredundant (nr) nucleotide database with an E-value cutoff of 1E-5.

## Results

### Population Structure

**Figure 1:**
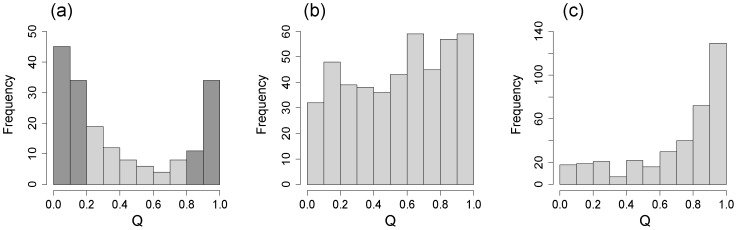
Frequency distributions of membership in the clusters defined by the reference population for *C. japonica*. (a) natural population (n = 181). Black bar represents the reference samples for STRUCTURE analysis. Q>0.8 indicates reference samples for Pacific Ocean side, while Q<0.2 those for Japan Sea side. (b) core collection (n = 456), (c) plus tree individuals in Kanto Breeding region (n = 367).

The genetic structure of the 367 plus trees in the Kanto Breeding Region was investigated using 292 SNP markers which were roughly evenly spaced over the genome. In STRUCTURE analysis, the highest likelihood was obtained when K was set to two, and using the method of Evanno *et* *al* [48], maximal ΔK occurred at K = 2, with the next largest peak at K = 4 in all two models (i.e. in the admixture model and the linkage model) and all three sample sets (the plus trees, the natural populations and the core collection) (Figure S1). We therefore examined the proportional membership of each individual in each cluster when K was 2. The frequency distributions of membership in the two clusters are shown in Figure 1. Incorporating prior information from the reference populations (membership coefficients of q_j_≥0.8) improved the genetic resolution and led to clear differences in the frequency distribution of Q between gene pools. The resulting frequency distribution was strongly bimodal for the natural population, with few individuals having Q values near 0.5 (Figure 1a), but it was not bimodal for the core collection, where many more individuals had intermediate Q values (Figure 1b). For samples from natural populations, membership of the two clusters showed a geographically structured pattern corresponding to the Japan Sea and Pacific Ocean sides of Japan. In the core collection samples, trees collected in the inland area had intermediate values of Q. In the plus tree samples from the Kanto Breeding Region, most of the samples showed a high membership coefficient for the Pacific Ocean side, and only a small proportion of them showed a high membership coefficient for the Japan Sea side. We chose K = 2 for the population structure matrix (Q-matrix) in the following association analysis. From the results of structure analysis, 195 and 116 samples were assigned to the Pacific Ocean side (with q_j_<0.8) and 37 and 80 samples were assigned to the Japan Sea side (with q_j_≥0.8) from the plus tree and core collection samples, respectively.

### Linkage Disequilibrium

**Figure 2:**
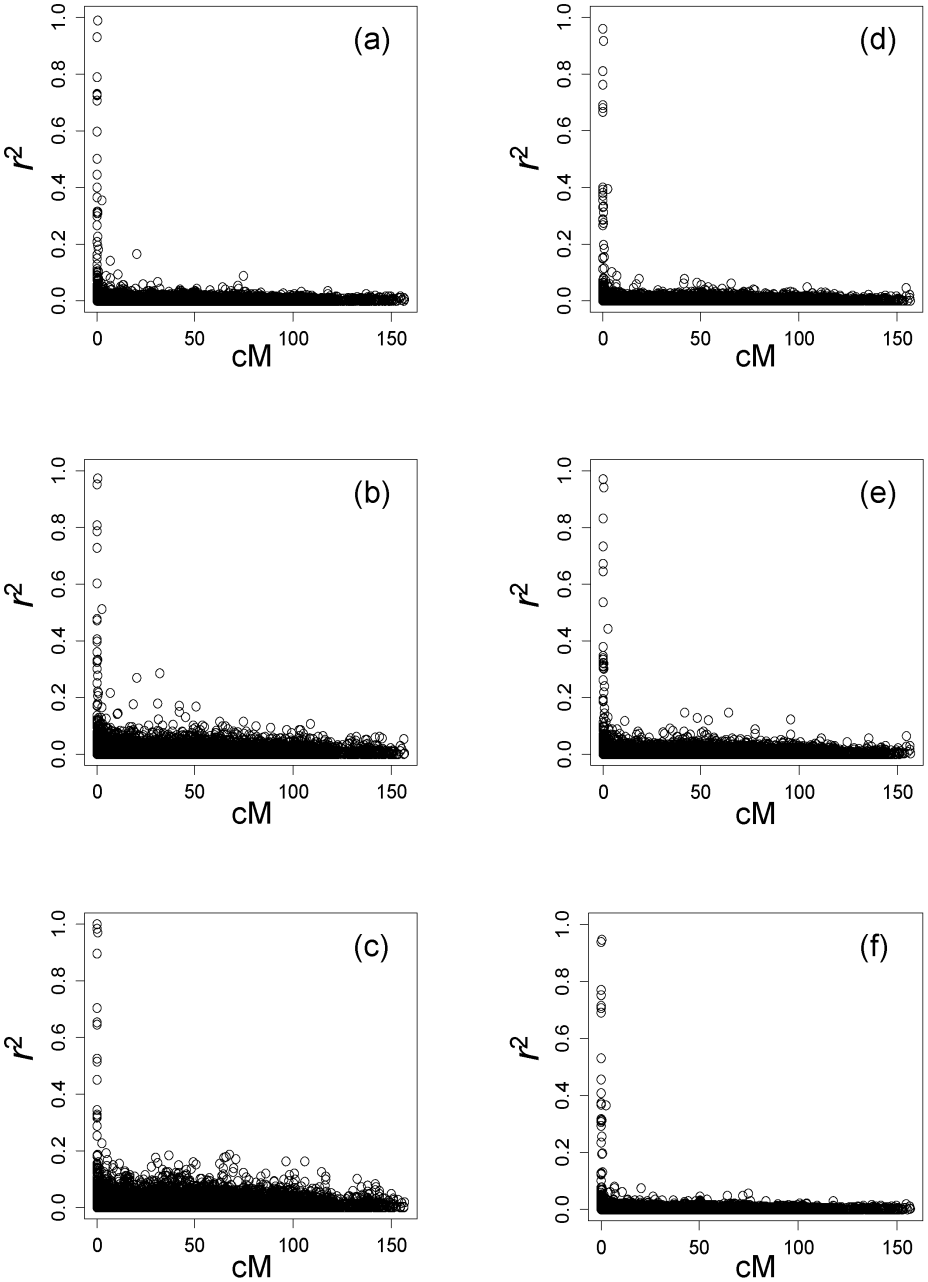
Linkage disequilibrium *r* ^2^, versus map distance in cM, for the *C. japonica* plus trees. (a) core collection (n = 456), (b) core collection (Pacific Ocean side (Q>0.8, n = 116)), (c) core collection (Japan Sea side (Q<0.2, n = 80)), (d) plus trees in Kanto Breeding Region (n = 367), (e) plus trees in Kanto Breeding Region (Pacific Ocean side (Q>0.8, n = 195),all samples (core collection and plus trees in Kanto Breeding Region (n = 731)).

**Figure 3:**
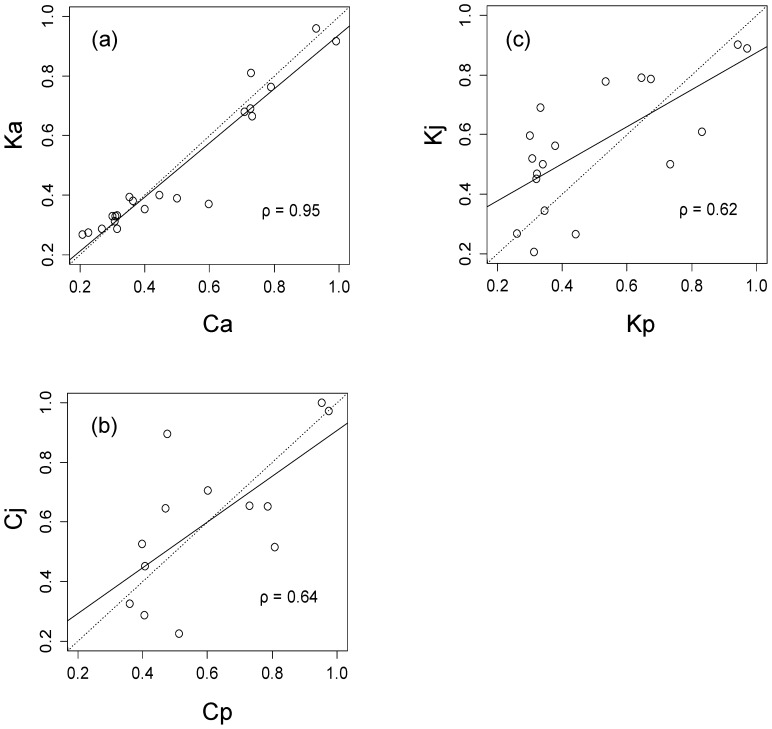
The correlation of significant *r* ^2^ values (fdr = 0.05, *r* ^2^>0.2) between two sample sets and two gene pools. (a) core collection (Ca) vs plus trees in Kanto Breeding Region (Ka), (b) core collection (Pacific Ocean side (Cp) vs Japan Sea side (Cj)), c) plus trees in Kanto Breeding Region (Pacific Ocean side (Kp) vs Japan Sea side (Kj)). The solid line represents linear correlation, and the dashed line represents line of identity. ρ is the spearman’s correlation coefficient.

The pairwise LD between the plus trees that we investigated was evaluated for all the polymorphic loci on the same chromosome (Table 1). Among all the samples, the average *r* ^2^ was 0.004, and 6.16% of the pairwise LD comparisons were significant at the 1% probability level (FDR = 0.1). Focusing on the subdivision between gene pools identified by the structure analysis, we found a slightly higher level of significant LD for the gene pool of the Pacific Ocean side (5.03% (p<0.01; FDR = 0.1); average *r* ^2^ = 0.007 (n = 195)) compared to the gene pool of the Japan Sea side (2.25% (p<0.01; FDR = 0.1); average *r* ^2^ = 0.030 (n = 37)). In the core collection samples, 12.70% of the pairwise LD comparisons were significant with an average *r* ^2^ of 0.004. With respect to the subdivisions in the gene pools of the core collection samples, each sample showed a similar level of LD to that of the plus tree samples (3.95% (p<0.01; FDR = 0.1); average *r* ^2^ = 0.011 (n = 116) for the Pacific Ocean side, 2.82% at the 1% probability level (FDR = 0.1), average *r* ^2^ = 0.014 (n = 80) for the Japan Sea side. Although the level of LD was almost similar (13.8% (p<0.01; FDR = 0.1); average *r* ^2^ = 0.003 (n = 731)), when we pooled all samples of plus tree individuals and core collection excluding the overlapped samples. Figure 2 gives LD as a function of genetic distance, showing that LD decays rapidly within approximately 1 cM in all samples (Figure 2 (a)-(f)). The pairs of SNPs showing significant LD were mostly the same for the plus trees and the core collection, and their *r* ^2^ values were strongly correlated between samples and gene pools (Figure 3).

**Table 1:** Linkage disequilibrium (LD) pair-wise pattern of *C. japonica* plus trees.

Sample type	Subsets	N	Mean *r* ^2^ ofall pairs	Marker pairs with signficant LD/total markerpairs (%) (p<0.01, FDR = 0.10)
plus trees in Kanto Breeding Region	Pacific Ocean	195	0.007	5.03
	Japan Sea	37	0.030	2.25
	All	367	0.004	6.16
core collection	Pacific Ocean	116	0.011	3.95
	Japan Sea	80	0.014	2.82
	All	456	0.004	12.70
All (excluding the overlapped samples)		731	0.003	13.80

### Association Analysis

**Figure 4:**
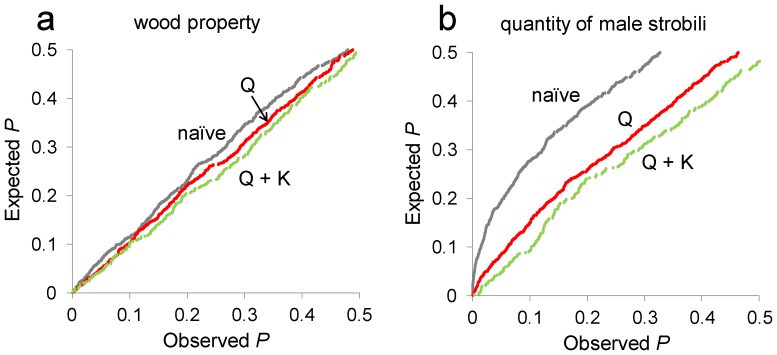
Comparison of models applied to *C. japonica* quantitative traits. Evaluation of the model type I error rates using random SNPs for wood property (a), quantity of male strobili (b). The cumulative distributions of observed *P-*values are presented for the naïve model, the Q model and the Q+K model.

Figure S2 is a plot of scaled phenotypic distance vs. genotypic distance for all marker pairs. The phenotypic distance matrix was estimated as all pairwise absolute differences between 367 genotypes. The genetic distances were calculated based on the manhattan distances. Not surprisingly, clones with similar genotypes have generally similar phenotypes; there are no points in the upper-left corner of the plot (especially for quantity of male strobili; Figure 4b). However, there are many cases of genotypically different clones with similar phenotypes. To evaluate the possibility of false positives in association models, we plotted expected *P*-values against observed *P*-values. The naïve model showed a higher deviation from the *y* = *x* line than Q and Q+K models, indicating that this method might detect a larger number of false positives than the others. The Q model was better than the naïve model but still showed the some deviation from the *y* = *x* line in comparison with the Q and Q+K models. According to Figure 4, the Q and Q+K models gave the smallest possibility of false positives among the models. In the naïve model, 17 and 87 significant associations were detected for wood property and quantity of male strobili, respectively (Table 2). To prevent false associations, we took into account the effects caused by population structure and kinship relationships in the association analysis. Under the model with population structure effects, the number of SNPs showing significant associations was reduced, especially with respect to number of male strobili. A total of 40 significant associations were detected under the model with population structure (Q model): 12 for wood property and 28 for quantity of male strobili. Under the model with the effects of population structure and kinship relationship (Q+K), 5 SNPs were significant for wood property and 1 for number of male strobili (Table 3). All the significant SNPs detected in Q+K model were also detected in the other two models. Of the 12 SNPs significant for wood property and the 28 SNPs significant for quantity of male strobili, according to the Q model, ten and 21, respectively, were also detected as significant in the naïve model. Some of the significant markers showed the same level of association in all the models, while in other cases markers were identified with different levels of significance by different models.

**Table 2:** Summary of models and number of significant loci at a nominal 1% level.

Model	Description	wood quality	quantity of male strobili
naïve	Simple test of association with no correction for population structure	17	87
Q	Inferred population structure as cofactor, i.e., structured association	12	28
Q+K	Mixed model with inferred population structure and kinship as fixed effect	5	1

**Table 3:** SNP locus annotations and significance values for wood property and quantity of male strobili.

Trait	SNP Locus	SNP	SNP type	Chromosomeposition	*P value*	FDR*Q value*	Markereffect	MAF	Annotation	*E-value*
wood property	gSNP01986	[A/T]	5′UTR	–	8.81E-05	0.091	0.047	0.334	microtubule-associatedprotein RP/EBfamily member 1-like	2.95E-84
	gSNP04252	[T/C]	synonymous	5	1.89E-03	0.976	0.030	0.175	lecithin cholesterolacyltransferase-like protein	1.81E-35
	gSNP03140	[A/C]	5′UTR	11	4.43E-03	1.000	0.025	0.269	RNA-binding protein40-like	2.89E-25
	gSNP01022	[A/G]	synonymous	10	4.51E-03	1.000	0.025	0.269	cytochrome P450	2.07E-48
	gSNP01196	[T/C]	intron	–	9.98E-03	1.000	0.020	0.026	fatty acyl-CoA reductase	3.94E-45
male strobili	gSNP00856	[T/C]	synonymous	–	2.73E-03	1.000	0.022	0.155	CLIP-associating protein	8.76E-25

Four out of the six SNPs showing significant associations in the Q+K model have been mapped onto the *Cryptomeria japonica* linkage map [44]. The SNPs gSNP04252, gSNP03140, gSNP01022 and gSNP01196 are located on linkage groups 5, 11, 10, and 7, respectively. These four SNPs were significantly associated with wood property. The remaining two of the six significant SNPs were not mapped onto the linkage map because they did not segregate in the mapping population. The proportions of variance explained by the four SNPs ranged from 2.0% for gSNP01196 to 4.7% for gSNP01986. In total, the four SNPs explained 14.8% of the phenotypic variation observed in the wood property trait. On the other hand, gSNP00856 explained only 2.2% of the phenotypic variation observed for the quantity of male strobili. Blastx homology searches identified known genes to which the six SNPs showed sequence similarity. For example, a microtubule-associated protein RP/EB family member appeared to be related to the wood property trait and a CLIP-associating protein to associate with quantity of male strobili (Table 3). After adjusting the significance threshold (to 10% of the false discovery rate) to take into account multiple testing, only one locus (gSNP01986) detected for the wood property trait was significant.

## Discussion

### Population Structure

Population structure can have a huge impact on the outcome of association studies, resulting in false positive associations [43], [59], and it is recommended that information about population structure be included in the statistical model used for GWAS as a factor affecting phenotypic variation [52], [60]. Crop species generally have highly genetically structured populations, as reported for maize [61], wheat [62], rice [63] and barley [64]. The population structure of these crops is thought to be the consequence of multiple events, e.g. modalities of domestication as shown in maize [65] and/or geographically diverse origins as demonstrated in rice [66], and to high levels of genetic differentiation between populations due to self-pollination breeding systems. In contrast, conifers generally have low levels of population structure. For example, the average level of background population structure identified by neutral loci such as microsatellite markers was low in *Pinus taeda* [9] and *Pinus sylvestris* [67]. This low level of structure is also reflected in the small proportion of variation found between populations at the allozyme level, which is often less than 5% [68], and at the nucleotide level as reported for *P. taeda* [69] and *P. sylvestris* [70]. In the discussion of a recent study on *C. japonica*, Tsumura *et* *al* [27] suggested that the genetic differentiation among 14 natural populations was also very low (*F*_ST_ = 0.0391). In the present study, however, the results of STRUCTURE analysis indicated that the samples of *C. japonica* taken from across its geographical range were clearly divided into two clusters, in the case of the core collection as well as in a natural population which was analyzed by Tsumura *et* *al* [27]. The distribution of membership coefficients between the two clusters, i.e. the Q values, reflected the geographical structure along the Japan Sea and Pacific Ocean sides of Japan, and may correspond to the two main varieties of *C. japonica*: “Omote-sugi” on the Pacific Ocean side of Japan and *C. japonica* var. *radicans* “Ura-sugi” on the Sea of Japan side [23], [27]. The Q values reveal clear bimodality in the natural populations, with few individuals having Q values near 0.5. In the core collection samples, in contrast, the Q values did not show a bimodal distribution, and many more individuals had intermediate Q values. This difference in the distribution pattern of Q values between the natural population and the core collection may result from the sampling schemes used. The natural populations are discontinuous and scattered in limited areas along the coastline [27], whereas the core collection individuals were sampled from various regional areas including inland populations (Miyamoto *et* *al*, in preparation). On the other hand, the plus tree samples subjected to association analysis had genetic backgrounds from the Pacific Ocean side, except for a few samples that clustered strongly with trees from the Japan Sea side (Figure 1-c), suggesting the importance of incorporating population structure effects in the association analysis. Although *C. japonica* is historically the most common and important species used for reforestation in Japan, the domestication and breeding of this species is still in its infancy, and selection of plus trees from the second generation has been initiated only recently [26]. Our results show that the plus tree populations have not suffered from diversity losses caused by a domestication bottleneck [71], and they retain the same allelic diversity as that of natural populations (Figure 1, Table S1).

### Linkage Disequilibrium

Detailed knowledge about the extent of LD in a population of breeding lines and cultivars is important when considering the future potential of GWAS in a target species [72]. In the present study, we evaluated the extent of LD in 367 *C. japonica* plus trees using 1,032 genome-wide markers. The overall LD measured in our samples was very low, and it decayed quickly within a few cM. The LD coefficient, *r* ^2^, summarizes both recombination and mutation history [73]. One important factor that can lead to low LD is the mating system of the species. An autogamous crop has a high LD because it offers no opportunities for new recombinants to be generated [74]. On the other hand, out-crossing leads to decreased LD because of the creation of new recombination. In predominantly selfing species like *A. thaliana* and rice, LD extends over large physical distances, for example >150 kb in *Arabidopsis* [74] and ∼100 kb in rice [75], whereas in outcrossing maize, the LD declines to negligible levels within 1 kb [76]. Conifers are predominantly allogamous species and the gene flow through pollen is highly efficient. Collectively, these life history traits lead to large effective population sizes in many commercially important conifer species, for instance *P. taeda*, *P. sylvestris*, *Pseudotsuga menziesii*, *Picea abies* and *C. japonica*. These characteristics would be expected to result in low LD due to high recombination rates at the population level. This prediction agrees with empirical data for several conifers, where relatively rapid decay of LD within genes (over a distance of 200–1500 bp) has been observed [77]–[80]. The rapid decay of LD observed in the present study, which is consistent with the prevailing outcrossing mating system and the high level of heterozygosity of *C. japonica*, is similar to the results from other conifer species.

The LD detected could be the result of two processes, population admixture and population structure attributable to recent coancestry. The population genetics theory predicts that disequilibrium due to admixture should have declined to negligible levels for nonsyntenic markers, provided that the population was randomly mating and reasonably large [81]. Although the plus tree samples in Kanto region clustered mainly with trees from the Pacific Ocean side of Japan, it might be anticipated that differences in allele frequency between gene pools would contribute to LD. However, a closer examination of the data set suggests that admixture has not contributed to LD in this population. When we calculated *r* ^2^ from two gene pools separately, the magnitude of LD and its relationship with distance were almost identical to the patterns observed in Figure 2 and 3. Thus it seems that admixture of two gene pools made a negligible contribution to LD in this case.

The power of GWAS depends largely on the strength of LD [71], [82]. When LD extends further across the genome, DNA markers have higher LD with QTL, and it is easier to detect the association between phenotypic traits and markers that are in LD with QTL. However, higher LD also has a drawback: positional resolution, i.e. difficulties in assigning the association to a particular candidate gene or SNP underlying a quantitative trait. Given the population’s low LD, it can be inferred that a large number of markers will be necessary in order to identify the QTLs responsible for important traits. On the other hand, however, the low LD can reduce the occurrence of spurious associations which are possible due to extended LD and/or loci on different chromosomes [71], [83]. The results of the present study suggest that a larger number of markers will be required to explain all QTLs associated with important traits in *C. japonica,* although the relationship between physical distance and genetic map distances is not obvious.

### GWAS for Traits in *C. japonica*

Population structure can be the result of common ancestry of large groups of individuals leads to spurious associations, which can be controlled by using a structure matrix [43] in the association analysis. Cryptic relatedness which is due to recent common ancestry among smaller groups of individuals should also be controlled for in the association analysis, as this can have a confounding effect similar to that of population structure [84]. With this in mind, we used a mixed model proposed by Yu *et* *al* [52], which has been successfully implemented for many traits in many crops [85]–[87], including tree species [9]. In many cases, a combined structure and kinship approach has been successful in interpreting the results [86], [87]. However, since the power of a mixed model is dependent on phenotypes, markers, population structure and relatedness, we tested multiple models which have been shown to perform better than other models in some circumstances [4], [87]. To evaluate the possibility of false positives in association models, we plotted the expected *P*-values against the observed *P*-values and tested three different linear regression models (Figure 4). Ideally, the *P*-values obtained from a mixed model follow a uniform distribution (***y*** = ***x*** line) in a *P*-*P* plot [52], [87]. As expected, the naïve model, which did not control the effects caused by both population structure and kinship, showed the highest inflation of *P*-values (i.e., *P*-values were not uniformly distributed), and consequently gave the highest Type I error. Controlling for population structure using the Q model yielded a considerable improvement over the naïve model, but a slight inflation of *P*-values still occurred. On the other hand, the Q+K model showed a good approximation to a uniform distribution of *P*-values. This may indicate that familial relatedness (i.e., kinship) should also be taken into account in the model used for association mapping. In sorghum [85], [88] and *Arabidopsis* [17], models accounting for both population structure and kinship performed better than those that controlled solely for Q or K. Our results also showed that the models that account for both population structure and kinship tended to perform better than other models for both wood property and quantity of male strobili (Figure 4).

However, the magnitude of improvement achieved by accounting for both Q and K was trait-dependent. The strong reduction in the false positive rate achieved by considering the Q matrix in the case of quantity of male strobili revealed that this trait was more strongly affected by population structure than was wood property. The number of loci showing significant association with quantity of male strobili was also dramatically reduced (from 87 to 17) after taking population structure into account, whereas for wood property the reduction was much smaller (from 17 to 12) (Table 2). For quantity of male strobili, after taking a consensus among the methods, only one locus (gSNP00856) was identified that was significant after accounting for population genetic structure but not after multiple testing correction (Table 3), and the effect attributed to the SNP allele was very small (2.2%). The large number of loci obtained from the naïve model applied to quantity of male strobili may include many false positives.

In outbreeding species, flowering of individual plants must be substantially synchronous within a local population to ensure mating success. Several data suggest that flowering time has evolved under selection for adaptation to local conditions in species such as *Arabidopsis* and maize [89]–[91]. In maize, in a large-scale study with high power, the effects of individual SNP on flowering time were very small, but whole genome associations accounted for most of the additive genetic variance [89]. The authors concluded that small-effect QTLs may permit adaptation to a wide range of environments by accumulation of alleles that consistently increase or decrease flowering time. *C. japonica* is distributed across many different environments in the Japanese Archipelago [92] and may be adapted to each local environment. In a previous study using 1,026 SNP loci, a large number of outlier and environment-associated loci were identified as potential local adaptation genes [27]. Our results suggest that the quantity of male strobili may be under selection and show polygenic inheritance.

In contrast, for wood property, a total of 5 loci were significantly associated with the trait after accounting for population genetic structure and kinship relatedness. Putative functions for these loci were revealed by BLASTx searches. For example, gSNP01986 is within a gene encoding a microtubule-associated protein, which may influence the stability of cortical microtubules and is believed to regulate dynamic cytoskeletal changes in plants [93]. One of the genes (gSNP01022) significantly associated with wood property showed similarity to the cytochrome P450 family (gSNP01022). Some of the gene products in this family are thought to be involved in lignin formation, although the gene including gSNP01022 showed greatest similarity to CYTOCHROME P450, FAMILY 704, which is not involved in lignin formation. The gene including gSNP01196 showed high similarity to fatty acid reductase 5 from *Arabidopsis thaliana*, and the gene involved in suberin synthesis. Although these putative functions may lead to explanations for the associations between these SNPs and wood property, further analysis will be needed to unveil the nature of the associations that we detected. So far, only limited studies on candidate gene associations have been conducted with respect to wood-quality genes in *P. taeda* [9], *P. radiata* [94] and *P. glauca* [12]. These candidate gene-based studies have, in general, confirmed the previous results of QTL mapping, i.e. the effects of individual loci on quantitative traits are mostly small, and the total effects detected are still far from accounting for all of the heritability of a given trait. In our study, the effects attributed to SNP alleles were also small (Table 3), in line with association studies in other tree species [9], [10], [95]–[97], and consistent with their quantitative nature.

### The Potential of GWAS for Conifers

On the basis of the rapid decay of linkage disequilibrium in conifers, Neale and Savolainen [7] concluded that genome-wide association studies would not be possible in these species because of the enormous density of SNP markers required (see also [98]). Currently a candidate gene approach is therefore more commonly used (e.g. [9]–[12]) to identify important loci such as those controlling wood property in conifers. However, recently, more extensive linkage disequilibrium has been found in some genes in *Pinus sylvestris* [99] and *Pinus taeda* [100]. Furthermore, Moritsuka *et* *al* [101] reported that LD was extensive and did not decay even at a distance of 100 kb in non-coding regions of the *C. japonica* genome. In many plants, recombination rates vary between different genomic regions (reviewed in [102]). In our study, we used 1,032 SNPs for GWAS and successfully detected 6 loci underlying important traits in our species. The number of SNP markers required may not be so large if most of the genome is segregating as blocks, as found by Moritsuka *et* *al* [101]. Thus, genome-wide association studies may also be feasible in conifers even though their genomes are generally large [103].

Recently, genomic selection (GS) has attracted increasing attention in the animal and plant breeding communities [104], [105]. The potential of GS has been discussed in the context of forest tree breeding [106]–[110]. In GS, a large number of DNA markers is required for modeling the relationship between multi-genotypes and phenotypic values of target traits. Although identifying causal polymorphisms is not necessary for genomic selection [104], [107], the genome-wide DNA markers developed for GWAS can be used directly for GS modeling. In forest tree breeding, GS is expected to be one of the most efficient selection methods because it can circumvent some issues such as the long generation time, high heterozygosity, and huge body size of these species. GWAS can detect unknown causal genes which could be used for MAS whereas GS cannot. The difference between MAS and GS is that MAS only utilizes the SNPs that are significant in a GWAS, whereas GS uses a genome-wide panel of dense markers so that all QTL are expected to be in LD with at least one marker. If it is true that most complex traits are controlled by many polymorphisms with small effect, GS has very large potential for future conifer breeding because GS could include all QTL effects in the prediction model. A candidate gene approach would be still be suitable for traits controlled by major genes, whereas GS has the advantage of being able to deliver superior phenotypes even for traits of a polygenic nature. Thus, GWAS and GS can each compensate for the other’s deficiencies, and both approaches are likely to be useful in conifer breeding. Genotyping based on next generation sequencing has become popular [111]–[114], and this approach is soon likely to overcome the cost problem currently inherent in genotyping a large number of DNA markers over a large number of samples.

## Supporting Information

Figure S1 **Plot of mean posterior probability (LnP(D)) values (open circles) per clusters (K), based on 10 replicates per K, generated by the STRUCTURE program (Pritchard et al., 2000), and delta-K analysis (filled squares) of LnP(D), according to Evanno et al., (2005).** (a) plus trees in Kanto Breeding Region (b) natural population (c) core collection.
(TIF)

Figure S2 **Relationship between phenotypic distance and marker distance.** a) wood property, b) quantity of male strobili
(TIF)

Table S1 **Genetic diversity statistics for plus trees and natural population in** ***C. japonica.***
(XLS)
