# Supplementary figures and images for: Demonstration of Genome-Wide Association Studies for Identifying Markers for Wood Property and Male Strobili Traits in Cryptomeria japonica
Source: PLoS One. 2013 Nov 19;8(11):e79866. doi: 10.1371/journal.pone.0079866 (PMC3833940; doi:10.1371/journal.pone.0079866)

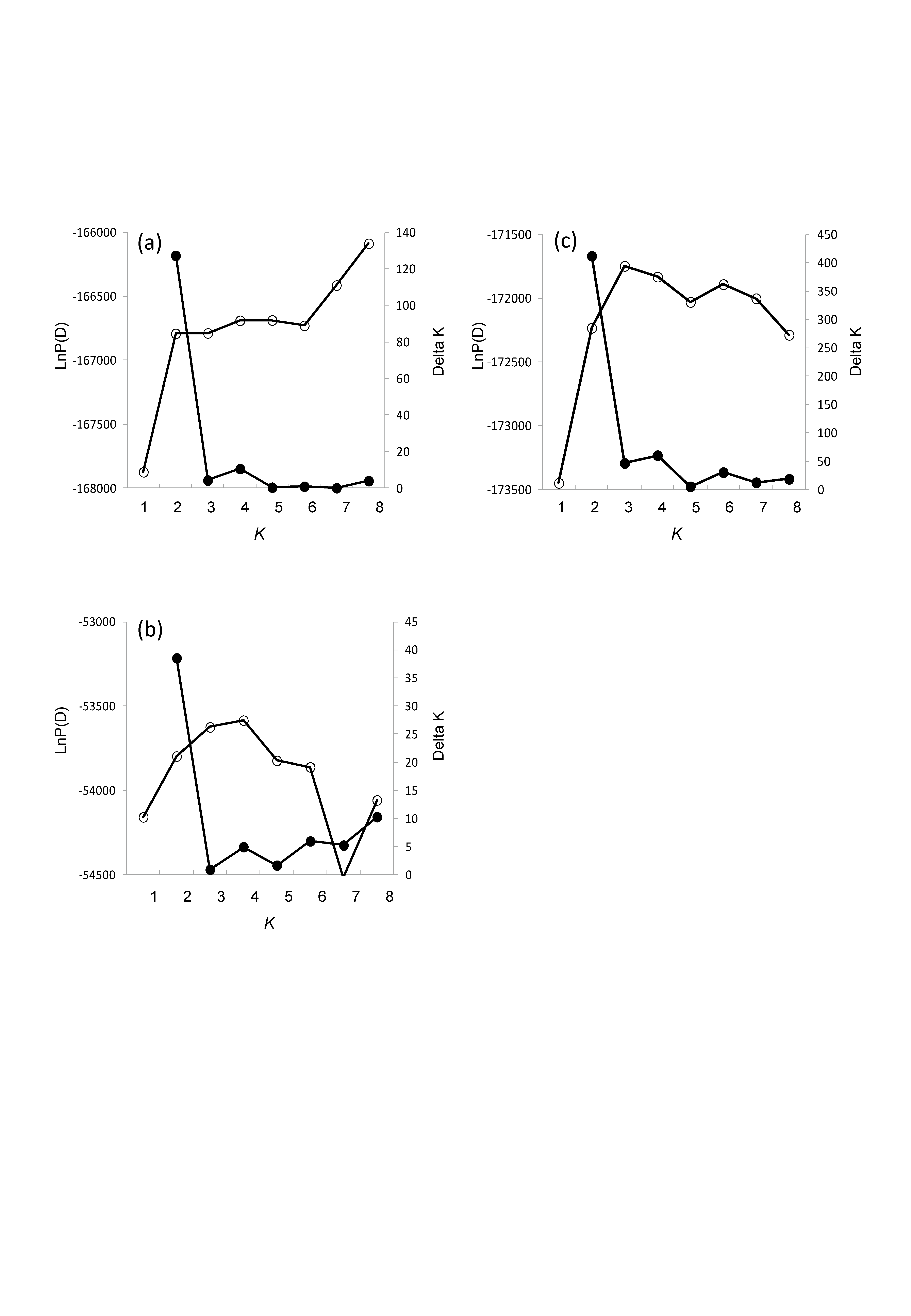

Supplement: Figure S1 — Plot of mean posterior probability (LnP(D)) values (open circles) per clusters (K), based on 10 replicates per K, generated by the STRUCTURE program (Pritchard et al., 2000), and delta-K analysis (filled squares) of LnP(D), according to Evanno et al., (2005). (a) plus trees in Kanto Breeding Region (b) natural population (c) core collection. (TIF) [file pone.0079866.s001.tif]
